# Congruent Deep Relationships in the Grape Family (Vitaceae) Based on Sequences of Chloroplast Genomes and Mitochondrial Genes via Genome Skimming

**DOI:** 10.1371/journal.pone.0144701

**Published:** 2015-12-14

**Authors:** Ning Zhang, Jun Wen, Elizabeth A. Zimmer

**Affiliations:** Department of Botany, National Museum of Natural History, MRC 166, Smithsonian Institution, Washington, DC, 20013–7012, United States of America; Universidad Miguel Hernández de Elche, SPAIN

## Abstract

Vitaceae is well-known for having one of the most economically important fruits, i.e., the grape (*Vitis vinifera*). The deep phylogeny of the grape family was not resolved until a recent phylogenomic analysis of 417 nuclear genes from transcriptome data. However, it has been reported extensively that topologies based on nuclear and organellar genes may be incongruent due to differences in their evolutionary histories. Therefore, it is important to reconstruct a backbone phylogeny of the grape family using plastomes and mitochondrial genes. In this study, next-generation sequencing data sets of 27 species were obtained using genome skimming with total DNAs from silica-gel preserved tissue samples on an Illumina HiSeq 2500 instrument. Plastomes were assembled using the combination of *de novo* and reference genome (of *V*. *vinifera*) methods. Sixteen mitochondrial genes were also obtained via genome skimming using the reference genome of *V*. *vinifera*. Extensive phylogenetic analyses were performed using maximum likelihood and Bayesian methods. The topology based on either plastome data or mitochondrial genes is congruent with the one using hundreds of nuclear genes, indicating that the grape family did not exhibit significant reticulation at the deep level. The results showcase the power of genome skimming in capturing extensive phylogenetic data: especially from chloroplast and mitochondrial DNAs.

## Introduction

Vitaceae is an economically important plant family, containing the fruit species, the grape (*Vitis vinifera*), that can be eaten as fresh fruit or made into juice, wine, jelly, and raisins. Some other Vitaceae species, such as *Parthenocissus tricuspidata* (Boston ivy) and *P*. *quinquefolia* (Virginia creeper), are commonly used as ornamentals. About 900 species from 15 genera are documented in the grape family [1]. Except for the temperate species in the genera *Ampelopsis*, *Causonis*, *Nekemias*, *Parthenocissus*, and *Vitis* (~ 135 temperate species in the five genera), most species in the family are distributed in tropical and subtropical regions as woody or herbaceous climbers or rarely shrubs [2–8].

The molecular phylogenetic analyses of Vitaceae initially were studied using several plastid genes, such as *rbcL*, the *trnL*-*F* intron and spacer, the *atpB*-*rbcL* spacer, *rps16*, and the *trnC*-*petN* spacer [9–14]. The nuclear ribosomal ITS, and the nuclear gene *GAI1* (*GA INSENSITIVE 1*) that encodes one regulator of gibberellins were also used for resolving the Vitaceae phylogeny [10, 15]. Although not completely resolving the deep phylogeny of the Vitaceae, the phylogenetic study with the most comprehensive taxon sampling in the family by Ren et al. [14] identified five major clades in the family, i.e., the *Ampelocissus*-*Vitis*-*Nothocissus*-*Pterisanthes* clade, the *Parthenocissus*-*Yua* clade, the core *Cissus* clade, the *Cayratia*-*Cyphostemma*-*Tetrastigma* clade (hereafter called the CCT clade), and the *Ampelopsis*-*Rhoicissus*-*Clematicissus* clade. While taxa of three of the five major clades possess 5-merous flowers, taxa of the *Cissus* clade and the CCT clade have 4-merous flowers. However, because of the uncertain deep relationships, whether the 4-merous flower originated once or multiple times was difficult to determine, especially given the merosity of the outgroup Leeaceae species’ being mostly 5, but with 4 also present in some taxa.

Wen *et al*. [5] reconstructed a deep phylogeny of the grape family using 417 single-copy nuclear genes retrieved from transcriptomes of 15 Vitaceae species and the published genome of the wine grape [16], *V*. *vinifera*. In this case, a highly supported deep phylogeny was recovered for Vitaceae. In this topology, the *Ampelopsis*-*Rhoicissus* clade is the earliest divergent lineage and is characterized by having 5-merous flowers. The *Vitis*-*Ampelocissus* clade and the *Parthenocissus*-*Yua* clade are sister groups, and they possess 5-merous flowers. The CCT and the *Cissus* clades are sister to each other and taxa of both clades possess 4-merous flowers, suggesting a single origin of 4-merous taxa in the grape family.

It has been commonly reported that the evolutionary history of nuclear genes and that of organellar genes may be different due to incomplete lineage sorting [17] and/or reticulate evolution (hybridization, introgression and allopolyploidy) [18, 19]. Moreover, only 15 species were sampled in the Wen *et al*. phylogenomic study [5], which might have produced systematic errors into the phylogenetic analysis due to sparse sampling [20]. Since using a few plastid genes had not resolved the deep phylogenetic relationships, we chose to produce a deep phylogeny for Vitaceae using complete chloroplast genomes as well as selected mitochondrial (mt) sequences from 28 species (27 species of Vitaceae plus an outgroup species from Leeaceae). Reference chloroplast and mitochondrial genomes of *V*. *vinifera* were included in our analyses [21, 22].

With the recent rapid development of next-generation sequencing (NGS) tools, it has become efficient and cost-effective to obtain genomic sequence data and mine markers for plant phylogenetic analyses [23–29]. The genome skimming approach [19, 23, 30, 31] is a rapid and cost-effective strategy for generating phylogenetically informative data via low- to high-density shotgun sequencing of total genomic DNA and was chosen for the study because it generates large phylogenetic data sets and is bioinformatically straight-forward. Genome skimming was developed initially to obtain phylogenetic informative sites from nuclear ribosomal, mitochondrial and chloroplast DNA markers because there are hundreds to thousands of copies in one plant cell and can be easily obtained with a low degree of sequencing coverage [32]. Genome skimming has been applied successfully in various plant groups, for example, in the Sonoran Desert clade of *Asclepias* (Apocynaceae) [30], in the tropical tree family Chrysobalanaceae [33], in the palm family (Arecaceae) [34], and in ferns [35]. In this study, we intend to test: (1) whether the deep phylogeny of the grape family reconstructed using plastomes, the one using mitochondrial genes, and the one using hundreds of nuclear genes reported previously are congruent; and (2) whether ancient hybridization occurred in the deep evolution of the grape family.

## Materials and Methods

### Ethics Statement

No specific permits were required for the collection of samples as they were all grown in the greenhouse, which complied with all relevant regulations. None of the samples represents endangered or protected species (Table 1).

**Table 1:** Sampling design and information on the NGS and plastome data. All voucher specimens were deposited at the United States National Herbarium (US).

Species	Accession number	Data size (Gb)	Number of reads	Coverage of plastome	Plastome size (bp)
*Vitis flexuosa*	*Wen 12461*	3.52	23,496,856	1,689	160,964
*Vitis riparia*	*Wen 12695*	3.11	20,763,492	1,671	161,011
*Vitis vinifera*	N/A	N/A	NC_007957	N/A	160,928
*Vitis rotundifolia var*. *munsoniana*	*Wen 12195*	2.12	14,118,242	1,178	161,275
*Pterisanthes heterantha*	*Wen 11820*	2.95	19,648,540	773	155,700
*Ampelocissus ascendiflora*	*Wen 11822*	2.97	19,825,858	880	155,686
*Parthenocissus vitacea*	*Wen 11976*	2.70	17,988,788	679	161,640
*Parthenocissus heptaphylla*	*Wen 11985*	2.80	18,672,228	1,133	161,467
*Tetrastigma sp*. *nov*.	*Wen 2011-fujian*	3.12	20,780,600	1,064	156,880
*Tetrastigma raffesiae*	*Wen 11824*	2.57	17,137,048	161	160,089*
*Tetrastigma lawsonii*	*Wen 11680*	2.67	17,809,412	602	160,232*
*Tetrastigma voinierianum*	*Wen 12–012*	3.01	20,078,184	142	159,986*
*Cayratia japonica*	*Wen UNI68*	2.71	18,063,906	829	158,240*
*Cyphostemma juttae*	*Wen 2010–093*	2.31	15,375,710	480	159,245
*Cyphostemma humile*	*Wen 2010–090*	2.15	14,353,720	136	158,800
*Cyphostemma sandersonii*	*Wen 2010–094*	2.17	14,450,758	724	159,196
*Cyphostemma adenopoda*	*Wen UC Davis*	2.47	16,487,240	400	159,495
*Cissus trifoliata*	*Wen 11977*	2.35	15,641,708	2,006	160,220
*Cissus tuberosa*	*Wen 2010–091*	1.93	12,848,572	995	159,928
*Cissus microcarpa*	*Wen 11954*	2.80	18,638,058	307	160,390
*Cissus discolor*	*Wen 12–011*	3.11	20,737,692	953	158,713
*Cissus quadrangularis*	*2010–086*	3.08	20,537,768	2,309	160,382
*Cissus antarctica*	*Wen 2012–010*	2.84	18,913,388	595	161,706*
*Ampelopsis aconitifolia*	*Wen 2010–078*	3.62	24,125,332	1,883	162,649*
*Ampelopsis cordata*	*Wen 12620*	2.93	19,539,876	879	161,955*
*Rhoicissus digitata*	*Wen 2010–097*	2.51	16,705,996	681	160,619*
*Nekemias arborea*	*Wen 12005*	3.23	21,529,740	713	162,625*
*Leea guineensis*	*Wen 2010–095*	2.45	16,356,482	254	160,555*

*: small gaps were not bridged for this plastid genome

### Sampling and DNA extraction

To cover the major lineages of the grape family, 27 species were sampled in this study (Table 1). Total DNA was isolated from ~ 15 mg silica gel dried leaves with the NucleoSpin Plant II DNA extraction kit (Macherey-Nagel, REF 740770.250). Then those DNAs were sent to the Genomic Sequencing and Analysis Facility (GSAF), University of Texas, Austin, for library construction and sequencing. Paired-end reads of 2 x 150 bp for all 27 species were generated in a single lane on an Illumina HiSeq2500 instrument. All raw data have been deposited into the GenBank with the BioProject accession number being PRJNA298058.

### Assembly of plastid genomes

The raw data obtained from the GSAF was filtered using Trimmomatic version 0.32 [36] with default settings. The plastome sequence of the wine grape (*V*. *vinifera*) was downloaded from GenBank (NC_007957) and was used as the reference plastid genome. For each species, the plastome was assembled using both the reference genome and *de novo* methods. The reference-guided assembly was performed using Bowtie2 [37] implemented in the Geneious program package (R8 version) with default settings [38]. The *de novo* assembly was conducted with Velvet [39] implemented in Geneious with the K-mer ranging from 69 to 99. The best K-mer was determined with the Velvet Optimiser implemented in Geneious with the K-mer choice and the coverage optimization being Lcon and Lbp, respectively. To correct errors or ambiguities resulting from either assembly method, the consensus sequence obtained using the reference genome was extracted and then used as the reference sequence for mapping of contigs obtained by *de novo* assembly. Then the sequence of each plastid genome was adjusted manually based on alignment of the consensus sequence and contigs mentioned above.

### Assembly of genes of mitochondrial origin in the grape family

Goremykin *et al*. [22] sequenced the mitochondrial genome of the wine grape (GenBank accession number NC_012119). To obtain genes of mitochondrial origin in the other sampled taxa of Vitaceae, we mapped NGS data onto the published grape mitochondrial genome for each species and extracted the sequences of 16 regions (S2 Table). Complete mitochondrial genomes were not produced for the 27 taxa in this study, because they are significantly longer than the chloroplast genomes and evolve with frequent rearrangements and horizontal gene transfers [22] that make alignments and determination of homology difficult.

### Genome annotation and phylogenetic analyses

In total, the plastomes of 28 species were aligned using MAFFT [40] implemented in Geneious. With the annotation of the grape plastome available, we could easily predict the coding and noncoding regions for the other 27 species. The alignments of coding and non-coding regions of each gene and those for intergenic regions were then obtained separately.

Because the sequences of plastid tRNA and rRNA are highly conserved, we excluded them from further analyses. In addition, sequences of only one copy of the inverted repeat (IR) region were included for the analyses because gene sequences of the two IR copies are completely or nearly identical. With the exclusions, we have a data set of 79 protein-coding genes (S1 Table), and we concatenated all these genes into one matrix. We also reconstructed the phylogeny of Vitaceae using intergenic regions and introns, excluding those in one of the two IR regions as well. In total, 95 non-coding regions were used for phylogenetic analyses, and they were concatenated into one matrix. We also concatenated sequences of the 79 protein-coding genes and the 95 non-coding regions into one matrix and conducted the phylogenetic analyses.

For genes of mitochondrial origin, we selected and concatenated 16 regions (S2 Table) with a significant number of variable sites across Vitaceae for phylogenetic analyses. All gene sequence alignments were deposited into the Dryad with the DOI being 10.5061/dryad.d15v3.

Maximum likelihood (ML) and Bayesian trees were inferred with RAxML [41] and MrBayes 3.2.1 [42, 43], respectively, with concatenated matrix being partitioned by genes. For ML analysis, the model was specified as GTR + CAT; 100 fast bootstrap ML reps were performed. For Bayesian analysis, two runs with 4 chains were run for 1,000,000 generations, and the model was specified as GTR + G. The MCMC convergence in Bayesian inference was monitored by AWTY (http://ceb.csit.fsu.edu/awty). Trees were sampled at every 100 generations. The first 25% of trees were discarded as burn-in with the remaining trees being used for generating the consensus tree.

## Results and Discussion

### Strategies for assembling plastomes

Overall, we obtained 74 Gb of data generated in a single Illumina lane for the 27 species. The minimum and the maximum size of the NGS data was 1.93 (12,848,572 reads) and 3.62 Gb (24,125,332 reads), for *Cissus tuberosa* and *Ampelopsis aconitifolia*, respectively. The average size of the data set for each of the 27 species was 2.75 Gb (Table 1).

For the three *Vitis* species, we obtained nearly complete plastid genomes with only two small gaps in AT-rich regions, using *V*. *vinifera* as the reference. We were able to bridge the two gaps in the *Vitis* species by mapping contigs from *de novo* assembly onto the consensus sequence from the reference genome method. However, for species from other genera, there were many gaps and ambiguous sites in the assembled plastomes from the reference genome method. For example, there were 23 gaps in the initial plastome of *Pterisanthes heterantha*, one species from the sister lineage of *Vitis* assembled with the reference method, in spite of 773X mapping coverage (981,188 [number of reads mapping on the grape plastome] * 147 bp [average length of reads] /186789 bp [length of consensus sequence]). All gaps were bridged by using both reference and *de novo* assembly methods. Except for gaps, ambiguous sites generated using the reference genome assembly method were another source of errors. Nevertheless, in combination with the *de novo* assembly method, these ambiguous sites were all corrected in *Pterisanthes heterantha*. For plastome assembly at the family level, we found it necessary to interactively use both methods.

Another strategy we used for the plastome assembly was a successive reference approach. After obtaining a complete plastome with high quality from one species (for example, *Pterisanthes heterantha)*, we used it as the reference genome for assembling the plastome of its most closely related relative based on our taxonomic understanding of the family **(**i.e., *Ampelocissus ascendiflora*), rather than using that of *V*. *vinifera*. There were many more poorly mapped regions (16 versus 3) and ambiguous sites (1345 versus 107) in *Ampelocissus ascendiflora*, if *V*. *vinifera* was used as the reference genome.

After extensive comparative assemblies, we obtained 17 complete plastomes with no gaps and ten plastomes with one to three gaps only (Table 1), all of which were in intergenic regions. These gaps were excluded from further phylogenetic analyses to reduce the impact of missing data.

### Resolving the backbone of Vitaceae with chloroplast and mitochondrial data

**Fig 1:**
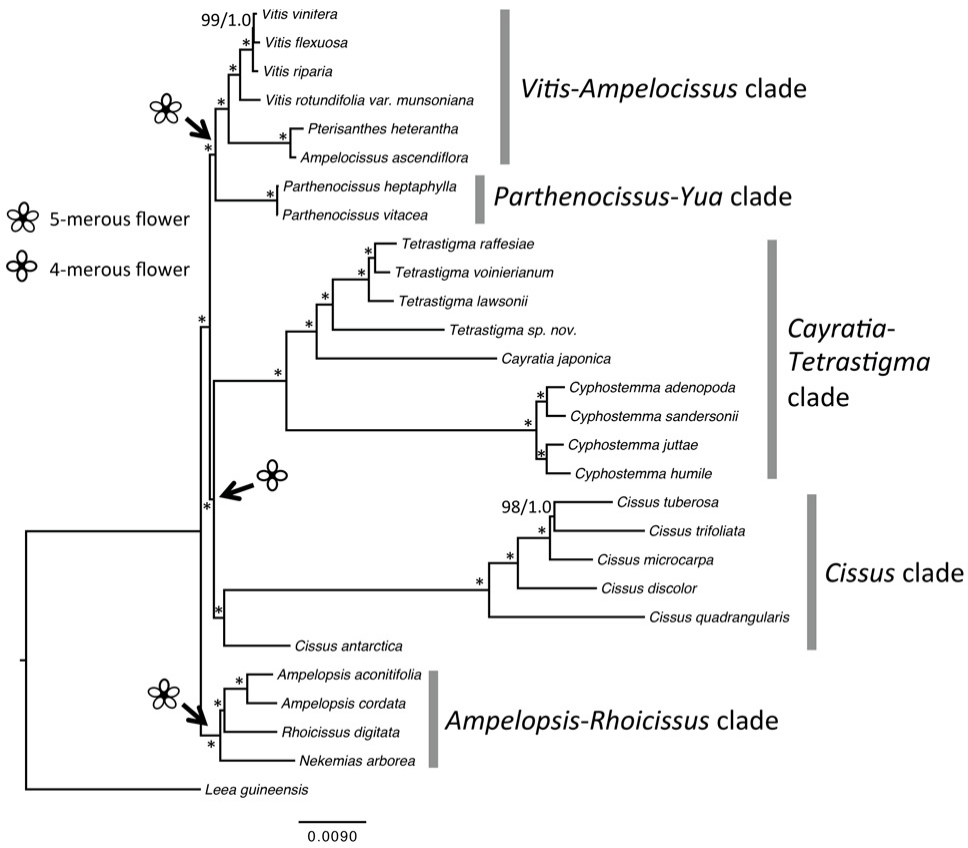
The backbone relationships of the grape family resolved by sequences of complete plastomes. The tree was reconstructed using RaxML and MrBayes with gene partitioning, which resulted in the same topology. Numbers associated with the branches are bootstrap value and posterior probabilities, with the asterisk indicating the node having a bootstrap value of 100% and a posterior probability of 1.0.

**Fig 2:**
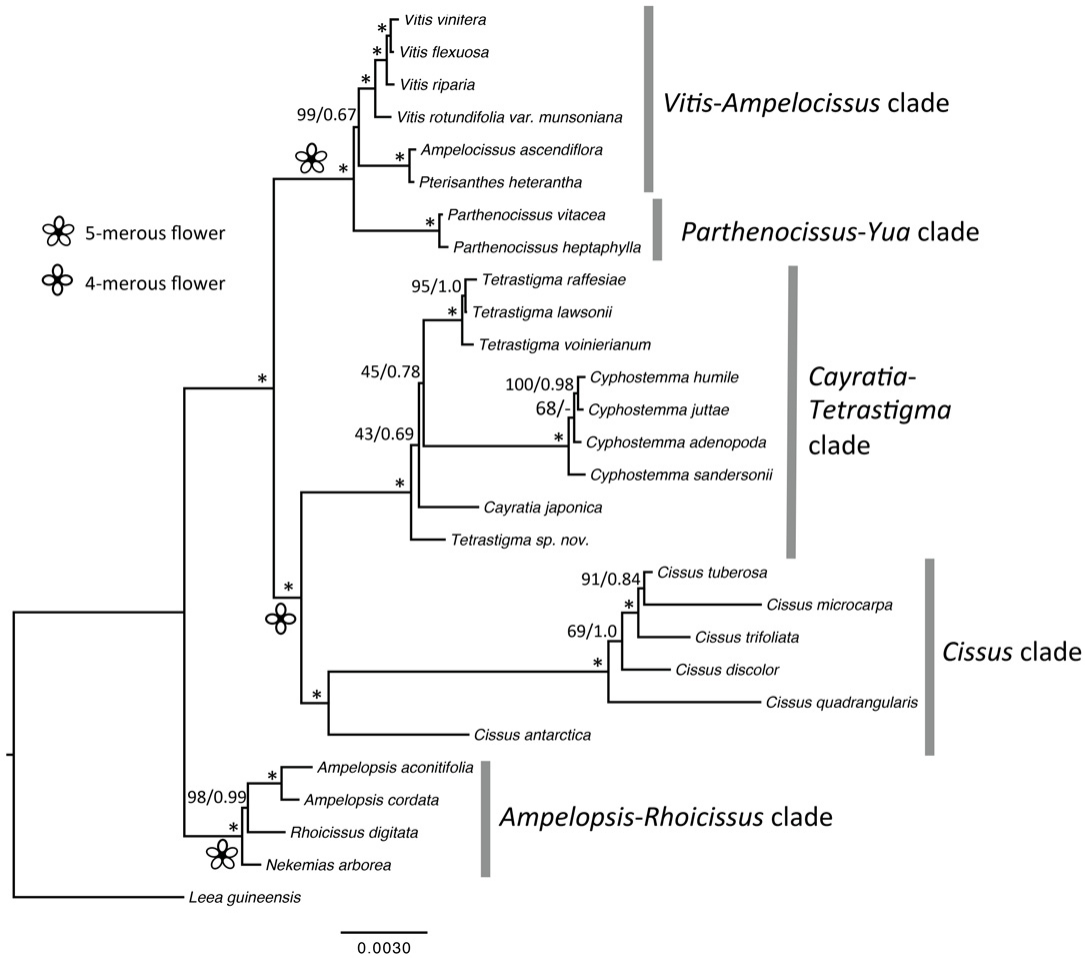
The backbone relationships of the grape family resolved using 16 mitochondrion origin regions. Numbers associated with the branches are bootstrap values and posterior probabilities obtained using RaxML and MrBayes, respectively. The asterisk indicates that the bootstrap value is 100% and the posterior probability is 1.0 at the node.

Plastid genomes represent an important source for characters to be used in plant phylogenetic analyses. To test if the topology using plastomes was congruent with the one using the hundreds of nuclear genes reported by Wen *et al*. [5], we used various analytical strategies to explore our plastome data. We first performed a partitioned analysis of the concatenated data of 126,683 aligned sites of the 28-plastome matrix by excluding one inverted repeat region for phylogenetic reconstruction. The ML and Bayesian topologies from this analysis (Fig 1) were congruent with the one generated from 417 single-copy orthologous nuclear genes with high support values. The topologies differed only in one node concerning the relationships of *Nekemias arborea* and *Rhoicissus digitata*. In the nuclear tree, *Nekemias* was sister to *Rhoicissus* with 100% bootstrap support, whereas *Nekemias* was sister to the clade of *Rhoicissus* and *Ampelopsis* s.s in plastome and mitochondrial topologies, with 100% and 98% bootstrap support, respectively (Figs 1 and 2). *Nekemias* was recently segregated from *Ampelopsis* s.l., because the latter was not monophyletic with *Rhoicissus* and the *Cissus striata* lineage nested within [6]. Either the nuclear or the organelle phylogenies still support the recognition of *Nekemias* as a distinct genus from *Ampelopsis*. With the consistent topology in the maternally inherited organelle phylogenies (Figs 1 and 2), the incongruence with the nuclear tree may be due to reticulate evolution, but we need to further explore the causes of such a topological difference.

We also tested the congruence of coding genes and noncoding regions in the plastomes, by analyzing the 79 protein-coding genes (S1 Fig) and the 95 non-coding regions separately (S2 Fig). Analyses from these two data sets generated the same topology with only slight differences in support values at some nodes.

In addition, we explored whether genes with different evolutionary rates may lead to congruent topologies. Evolutionary rates can be generally measured by sequence identity, i.e., the percentage of identical sites in all sites of an alignment, with faster evolving genes having lower identity scores. This strategy resulted in three data sets of 10 genes, 26 genes, 56 genes, corresponding with identity less than 80%, 85%, 90%, respectively (Table 2). Phylogenetic analyses were then performed using these three matrices. With the addition of more genes from low to high gene identity, the topology was almost identical except for the relationships among three *Cissus* species (S1 Fig). The 10-gene topology supported the sister relationship of *C*. *microcarpa* and *C*. *tuberosa* (bootstrap value 68), whereas the 26, 56 and 79-gene data sets all supported the sister relationship between *C*. *tuberosa* and *C*. *trifoliata* with bootstrap values of 41%, 23%, and 57%, respectively (S1 Fig).

**Table 2:** The four data matrices of the chloroplast protein coding genes based on gene identity.

Matrix based on gene identity thresholds	Number of genes	Size of alignment (bp)	Parsimony informative sites
Less than 80%	10	14,062	1,985
Less than 85%	26	25,883	2,880
Less than 90%	56	55,382	4,344
All 79 genes	79	69,800	4,832

We used mitochondrial genes as another source of phylogenetic data for Vitaceae. Since the mitochondrial genome of the wine grape is large, i.e., 773,279 bp [22], and 42.4% of chloroplast genome has been incorporated into the mitochondrial genome [22]. Therefore, it is impossible to assemble a complete mitochondrial genome using the genome skimming method because it is difficult to tease chloroplast-origin reads apart from mitochondrion-origin reads. Even though the Illumina sequencing generated 1.93–3.62 Gb data for each of the 27 species, and represented high-density coverage for a mt genome. We therefore mined the mitochondrion-specific genes conservatively to avoid potential error, using the grape mitochondrial genome as the reference. Because mitochondrial genes are relatively conserved, we selected 16 mt regions with a significant number of variable sites among taxa of the family for phylogenetic inference (S1 Table). The backbone topology reconstructed using these 16 mitochondrial regions (18,105 bp) is congruent with the one using the plastome data; however, the positions of *Tetrastigma* and *Cissus* can not be well resolved in the mt tree because only 3% of the total sites of 16 mitochondrial genes are parsimony-informative (Fig 2).

It has been reported extensively that phylogenies using genes from the three genomes (plastid, mitochondrial and nuclear) in plants may differ due to different evolutionary histories [17, 44–47]. Nevertheless, our study overall shows a congruent backbone phylogeny of the grape family using genes from the three compartments. Five major monophyletic lineages, i.e., the *Ampelopsis*-*Rhoicissus* clade, the *Cissus* clade, the *Cayratia*-*Cyphostemma*-*Tetrastigma* clade, the *Parthenocissus*-*Yua* clade, and the *Vitis*-*Ampelocissus* clade, were defined (Figs 1 and 2).

The position of *Cissus antarctica*, a species from Australia, has been difficult to resolve in previous studies [4, 10, 11]. It is resolved here using the complete plastomes as well as with the 16 mitochondrial genes. Both trees placed *Cissus antarctica* as the first diverged lineage of the *Cissus* clade. *Cissus antarctica* is closely related to four other *Cissus* species in Australia and New Guinea (*C*. *hypoglauca*, *C*. *oblonga*, *C*. *penninervis*, and *C*. *sterculiifolia*) and one species from the Neotropics (*C*. *trianae*) [4, 10, 48]. The position of this clade from our analyses thus supports treating the *Cissus antarctica* clade as part of *Cissus*, the largest genus of the family with about 300 species [1].

The *Cissus* clade and the CCT clade are sisters, and taxa of this large diverse clade is characterized by 4-merous flowers and well-developed thick floral discs [1]. This aspect of the topologies supports a single origin for 4-merous flowers from a 5-merous ancestor.

### Genome skimming as a valuable resource for plant phylogenetic analyses

Although most studies using a genome skimming approach produced low-density coverage of the whole genome of a species, large data sets of chloroplast, mitochondrial, rDNA, or even other nuclear genes have been obtained [30, 35, 49, 50]. With the high-density coverage produced in this study, we obtained all chloroplast protein coding genes and even assembled the complete plastomes as well as sequences of genes of mitochondrial origin using the genome skimming approach. As the sequencing costs using next-gen platforms have decreased rapidly in recent years, high-density genome skimming like this Vitaceae study is becoming affordable to colleagues in the systematics community. We thus advocate the high-density genome skimming approach, when possible, to potentially explore phylogenetic data from all three genomes, especially in cases where reticulation and horizontal gene transfer events may have been suspected. In the next phase of our study, we will continue to mine the nuclear data fraction from this Vitaceae Illumina run, especially for mining faster evolving regions/markers that our earlier transcriptome data largely did not cover, as the RNA-Seq approach produces sequences of only the coding regions of expressed genes [5].

Mitochondrial genes have been largely under-utilized in plant phylogenetic studies. Earlier plastome studies employed methods that isolated plastid DNA from fresh leaves via chloroplast enrichment using sucrose gradients [21], and mitochondrial genes were thus excluded in such studies. Another reason for the under-utilization of mt DNA comparisons is that plant mt DNA tends to evolve by structural rearrangements with slow rates of nucleotide substitution [51]. Furthermore, genome skimming can be tricky, because cp genes can be transferred into mt or vice versa [22, 52], so tests of whether a particular gene is most related to mitochondrial genes of reference species or to its chloroplast one may be useful. So far 295 mitochondrial genomes of 95 plant species have become available in GenBank. With the availability of a broad range of reference genomes, it is possible to mine mitochondrial-specific genes using the most closely related mitochondrial genome as the reference for many plant groups. For our analyses of Vitaceae, the mt genome of *V*. *vinifera* has been sequenced and assembled [22], which greatly facilitated the mining of genes of actual mitochondrial origin.

## Supporting Information

S1 Fig The phylogenetic relationships of Vitaceae reconstructed using four data sets. Numbers associated with the branches are bootstrap values obtained using 10, 26, 56 and 79 genes, corresponding to sequence identities lower than 80, 85, 90 and 100%, respectively. The asterisk indicates the bootstrap value as 100%. If each of the four bootstrap values from all matrices are 100%, one diamond is placed at the node. The dashes indicate incongruence of a relationship from the one using 79 protein-coding plastid genes (see text for details).
(EPS)

S2 Fig The phylogenetic relationships of Vitaceae reconstructed using the 95 non-coding plastid regions. Numbers associated with the branches are bootstrap values. The asterisk indicates a bootstrap value of 100%.
(EPS)

S1 Table The 79 protein-coding plastid genes used in the phylogenetic analyses of Vitaceae. (DOCX)

S2 Table The 16 regions of mitochondrial origin used in the phylogenetic analyses of Vitaceae. (DOCX)
