# Correction: Congruent Deep Relationships in the Grape Family (Vitaceae) Based on Sequences of Chloroplast Genomes and Mitochondrial Genes via Genome Skimming

**DOI:** 10.1371/journal.pone.0152059

**Published:** 2016-03-17

**Authors:** Ning Zhang, Jun Wen, Elizabeth A. Zimmer

There is an error in the fifth sentence of the Abstract. The correct sentence is: In this study, next-generation sequencing data sets of 27 species were obtained using genome skimming with total DNAs from silica-gel preserved tissue samples on an Illumina NextSeq 500 instrument.

There is an error in the second to last sentence of the “Sampling and DNA extraction” section of the Materials and Methods. The correct sentence is: Paired-end reads of 2 x 150 bp for all 27 species were generated in a single lane on an Illumina NextSeq 500 instrument.
