# Supplementary material for: Congruent Deep Relationships in the Grape Family (Vitaceae) Based on Sequences of Chloroplast Genomes and Mitochondrial Genes via Genome Skimming
Source: PLoS One. 2015 Dec 14;10(12):e0144701. doi: 10.1371/journal.pone.0144701 (PMC4682771; doi:10.1371/journal.pone.0144701)
Supplement: S1 Table — (DOCX) [file pone.0144701.s003.docx]

Table S1. The 79 protein-coding plastid genes used in the phylogenetic analyses of Vitaceae.

| **Gene** | **Length of alignment (bp)** | **Parsimony informative sites** | **PI_percentage (%)** |
| --- | --- | --- | --- |
| *rps12* | 258 | 1 | 0.39 |
| *ndhB* | 1,533 | 10 | 0.65 |
| *rpl2* | 825 | 9 | 1.09 |
| *rpl23* | 285 | 4 | 1.40 |
| *rps7* | 468 | 7 | 1.50 |
| *ycf2* | 6,993 | 119 | 1.70 |
| *petL* | 96 | 2 | 2.08 |
| *psbN* | 132 | 3 | 2.27 |
| *atpH* | 246 | 6 | 2.44 |
| *psbE* | 252 | 7 | 2.78 |
| *psbD* | 1,062 | 33 | 3.11 |
| *psbA* | 1,068 | 34 | 3.18 |
| *rps18* | 309 | 10 | 3.24 |
| *psbJ* | 123 | 4 | 3.25 |
| *psbF* | 120 | 4 | 3.33 |
| *petN* | 90 | 3 | 3.33 |
| *ndhC* | 363 | 13 | 3.58 |
| *ycf3* | 507 | 19 | 3.75 |
| *psaB* | 2,205 | 91 | 4.13 |
| *psbH* | 240 | 10 | 4.17 |
| *psbZ* | 189 | 8 | 4.23 |
| *psbL* | 117 | 5 | 4.27 |
| *psbB* | 1,527 | 70 | 4.58 |
| *atpI* | 744 | 35 | 4.70 |
| *petB* | 648 | 32 | 4.94 |
| *psbC* | 1,422 | 71 | 4.99 |
| *rpoC1* | 2,058 | 104 | 5.05 |
| *psaA* | 2,253 | 114 | 5.06 |
| *rpoB* | 3,214 | 163 | 5.07 |
| *psbT* | 115 | 6 | 5.22 |
| *psbI* | 111 | 6 | 5.41 |
| *rps8* | 405 | 22 | 5.43 |
| *rps2* | 711 | 39 | 5.49 |
| *ndhI* | 504 | 28 | 5.56 |
| *atpF* | 558 | 31 | 5.56 |
| *petD* | 484 | 27 | 5.58 |
| *petA* | 963 | 54 | 5.61 |
| *atpB* | 1,503 | 85 | 5.66 |
| *ndhJ* | 477 | 27 | 5.66 |
| *rpl14* | 369 | 21 | 5.69 |
| *psbM* | 105 | 6 | 5.71 |
| *psbK* | 192 | 11 | 5.73 |
| *ndhK* | 678 | 39 | 5.8 |
| *ycf4* | 567 | 33 | 5.8 |
| *petG* | 114 | 7 | 6.1 |
| *ndhG* | 534 | 34 | 6.4 |
| *psaC* | 246 | 16 | 6.5 |
| *ndhA* | 1,107 | 72 | 6.5 |
| *rps14* | 303 | 20 | 6.6 |
| *infA* | 240 | 16 | 6.7 |
| *atpE* | 402 | 27 | 6.7 |
| *rpoA* | 1,005 | 68 | 6.8 |
| *rpl16* | 408 | 28 | 6.9 |
| *accD* | 1,880 | 130 | 6.9 |
| *rps16* | 281 | 20 | 7.1 |
| *rbcL* | 1,428 | 102 | 7.1 |
| *ndhH* | 1,182 | 85 | 7.2 |
| *atpA* | 1,524 | 114 | 7.5 |
| *psaJ* | 132 | 10 | 7.6 |
| *ndhD* | 1504 | 115 | 7.6 |
| *rps4* | 612 | 48 | 7.8 |
| *rpl20* | 354 | 28 | 7.9 |
| *rpoC2* | 4,230 | 336 | 7.9 |
| *cemA* | 690 | 56 | 8.1 |
| *rps15* | 267 | 23 | 8.6 |
| *rpl36* | 114 | 10 | 8.8 |
| *rps11* | 417 | 38 | 9.1 |
| *clpP* | 591 | 54 | 9.1 |
| *ndhE* | 307 | 29 | 9.4 |
| *rps19* | 288 | 28 | 9.7 |
| *rps3* | 657 | 65 | 9.9 |
| *rpl33* | 201 | 22 | 10.9 |
| *ccsA* | 986 | 108 | 11.0 |
| *matK* | 1,515 | 174 | 11.5 |
| *ndhF* | 2,307 | 270 | 11.7 |
| *rpl32* | 174 | 21 | 12.1 |
| *rpl22* | 495 | 67 | 13.5 |
| *psaI* | 111 | 19 | 17.1 |
| *ycf1* | 6,105 | 1,146 | 18.8 |
