# Supplementary material for: Congruent Deep Relationships in the Grape Family (Vitaceae) Based on Sequences of Chloroplast Genomes and Mitochondrial Genes via Genome Skimming
Source: PLoS One. 2015 Dec 14;10(12):e0144701. doi: 10.1371/journal.pone.0144701 (PMC4682771; doi:10.1371/journal.pone.0144701)
Supplement: S2 Table — (DOCX) [file pone.0144701.s004.docx]

Table S2. The 16 regions of mitochondrial origin used in the phylogenetic analyses of Vitaceae.

| **Gene** | **Length of alignment (bp)** | **Parsimony informative sites** | **PI_percentage (%)** |
| --- | --- | --- | --- |
| *atp1* | 1,535 | 48 | 3.1 |
| *cox1* | 1,584 | 16 | 1.0 |
| *cox2* | 4,490 | 153 | 3.4 |
| *cox3* | 798 | 26 | 2.2 |
| *sdh4* | 360 | 18 | 5 |
| *cob2* | 1182 | 15 | 1.3 |
| *nadL* | 303 | 3 | 1.0 |
| *atp4* | 613 | 51 | 8.3 |
| *nadL*-*atp4* | 192 | 10 | 5.2 |
| *nad9* | 574 | 4 | 0.7 |
| *nad6* | 672 | 19 | 2.8 |
| *matR* | 1210 | 22 | 1.8 |
| *ccmB* | 621 | 46 | 7.4 |
| *ccmC* | 753 | 30 | 4.0 |
| *ccmFC* | 2,541 | 139 | 5.5 |
| *ccmFN* | 1,730 | 51 | 2.9 |
